# Ecological validity of cost-effectiveness models of universal HPV vaccination: a protocol for a systematic review

**DOI:** 10.1186/s13643-017-0409-7

**Published:** 2017-01-25

**Authors:** Giampiero Favato, Emmanouil Noikokyris, Riccardo Vecchiato

**Affiliations:** 1 Institute of Leadership and Management in Health, Kingston University London, London, UK; 2 Kingston Business School, Kingston University London, Kingston Hill, Kingston-upon-Thames, KT2 7LB London, UK

## Abstract

**Background:**

Sexually transmitted infection with high-risk, oncogenic strains of human papillomavirus (HPV) still induces a relevant burden of diseases on both men and women. Although vaccines appear to be highly efficacious in preventing the infection of the most common high-risk strains (HPV 6, 11, 16, 18), important questions regarding the appropriate target population for prophylactic vaccination are still debated. Models in the extant literature seem to converge on the cost-effectiveness of high coverage (>80%) of a single cohort of 12-year-old girls. This vaccination strategy should provide an adequate level of indirect protection (herd immunity) to the unvaccinated boys. This argument presupposes the ecological validity of the cost-effectiveness models; the implicit condition that the characteristics of the individuals and the sexual behaviours observed in the models is generalisable to the natural behaviours of the population.

The primary aim of this review is to test the ecological validity of the cost-effectiveness models of universal HPV vaccination available in the literature. The ecological validity of each model will be defined by the number of representative characteristics and behaviours taken into consideration.

**Methods:**

Nine bibliographic databases will be searched: MEDLINE (via PubMed); Scopus; Science Direct; EMBASE via OVID SP, Web of Science, DARE, NHIR EED and HTA (via NHIR CRD); and CINHAL Plus. An additional search for grey literature will be conducted on Google Scholar and Open Grey. A search strategy will be developed for each of the databases. Data will be extracted following a pre-determined spreadsheet and then clustered and prioritised: the main outcomes will report the inputs to the demographic and epidemiological model, while additional outcomes will refer to basic inputs to the cost-effectiveness valuation.

Each study included in the review will be scored by the number of representative characteristics and behaviours taken into consideration (yes or no) on both dimensions. Individual study’s scores will be plotted in a 2 by 2 matrix: studies included in the upper right quadrant will be defined as ecologically valid, since which both individuals’ characteristics and their sexual behaviours are representative.

**Discussion:**

The proposed systematic review will be the first to assess the ecological validity of cost-effectiveness studies. In the context of sexually transmitted diseases, when this condition is violated, an error in predicting the protective impact of herd immunity would occur. Hence, a vaccination policy informed on ecologically invalid models would potentially expose boys to a residual risk of contracting HPV-induced malignancies.

**Systematic review registration:**

PROSPERO CRD42016034145

**Electronic supplementary material:**

The online version of this article (doi:10.1186/s13643-017-0409-7) contains supplementary material, which is available to authorized users.

## Background

Sexually transmitted infection with high-risk, oncogenic strains of human papillomavirus (HPV) imposes a significant economic burden on public health providers. Much of the information on HPV centres on women since having the virus increases the risk of invasive cervical cancer. But HPV virus may cause other neoplastic benign and malignant lesions, equally affecting men and women [1]. The total lifetime costs attributable to non-cervical, HPV-induced diseases were found comparable for both sexes [2].

Over the last decade, the advent of the bivalent (Cervarix, GlaxoSmithKline) and the quadrivalent (Gardasil, Merck Sharp & Dohme) vaccine in addition to screening paradigms held promise for reducing the long-term, cumulative burden of HPV-induced diseases. The trade-off was an incremental annual investment in vaccination programmes. Under the constraint of scarce public resources, policy makers relied on economic valuation to inform the decision to add the HPV vaccination to existing immunisation programmes. According to previously published reviews, most cost-effectiveness studies in the extant literature evaluated women-only direct immunisation programmes [3–5]. The few studies which included males in the valuation of HPV vaccination seemed to reach controversial conclusions [6, 7]. A recent review focused on a range of values missing from most studies, such as indirect costs, right to access treatment and health equalities [8].

The unresolved dichotomy between public health leaders who advocate the urgency of vaccinating boys against HPV [9] and policy makers who are hesitant to allocate economic resources to universal (boys and girls) vaccination calls for a methodological review of the cost-effectiveness models used to inform the decisions of the latter.

The primary aim of this review is to test the ecological validity of the cost-effectiveness models available in the literature. This can be framed by asking if the models provide an acceptable representation of the intended reality on all relevant dimensions.

This systematic review protocol will adhere to the reporting guidelines of the Preferred Reporting Items for Systematic Reviews and Meta-Analyses Protocols (PRISMA-P) statement [10]. The PRISMA-P checklist can be found in Additional file 1.

### Ecological validity

Ecological validity has typically been taken to refer to whether or not one can generalise from observed behaviour in the laboratory to natural behaviours in the world [11]. In fact, ecological validity or the related idea of external validity has a long history in psychology and economics, with the issues central to this idea frequently and continuously debated [12].

The demand for ecological validity stems from the Brunswick’s concept of representative design, a construct used to assess whether an experiment or a model captures the relevant features of intended realities [13]. Using the replica model analogy [14], this can be framed by asking how well the model mimics reality on characteristics relevant to the theory. Central to representative design is the notion that any model can be thought as a sample of behaviours. For results to be generalisable, not only should attributes of participants be representative but also their expected behaviours in the specific task or situation. To do this, investigators need to specify the relevant characteristics and behaviour of the population related to the intended situation. In addition, sampling should take place on two dimensions. One involves the participants; the other concerns the situations or tasks with which the participants are confronted. Valid inferences can only be achieved by sampling in a representative manner on both dimensions.

Applied to cost-effectiveness of universal HPV vaccination, models chosen to inform public immunisation policies should take into account most of the dimensions relevant to the population (individuals susceptible to HPV infection) as well as to the specific situation (patterns of sexual mixing).

### Challenges of modelling sexual behaviour

Traditionally, patterns of sexual behaviour have been characterised by focusing on the individual as unit of analysis, e.g. by measuring an individual’s number of sexual partners. However, adopting a network approach to the epidemiology of sexually transmitted infections reveals that behaviours and sexual preferences may influence the spread of infection [15]. The ecological theory of infectious disease discusses the characteristics of the individuals and the patterns of the sexual mixing relevant to HPV infection [16]. Gender, age, ethnicity, sexual activity, rate of change of sexual partners and frequency of unprotected sex are characteristics representative of individuals at risk of infection [17–19]. Concurrent partnership and assortative mixing are relevant to the model’s representativeness of sexual mixing. Concurrent partnerships are those in which the same individual is involved in sexual partnerships occurring simultaneously, as opposed to “serial monogamy” where one partnership ends before another one starts. The proportion of concurrent partnerships effectively increases the risk of sexually transmitted infections in the population [20, 21]. The risk of infection varies according to whether partnerships are formed between people from similar (positive assortative) or different (negative assortative) prevalence and sexual activity groups. Sex with partners of the same sex (positive) [22], sex with older partners (negative) [23], sex with a foreign partner (negative) [24] and pay for sex (negative) [25] are the four behaviours which increase the heterogeneity in the population, since some susceptible individuals are more likely to acquire and transmit the infection. If the high-risk individuals mix randomly, the infection is more likely to spread [26].

Building upon the same methodological premise of ecology of infectious disease, the Natsal survey is aimed to understand the most current characteristics of sexual partners and partnerships in the general British population. Three Natsal surveys have taken place up to day: Natsal-1 in 1990–1991, Natsal-2 in 1999–2001 and Natsal-3 in 2010–2012. In the Natsal-3 [27, 28], the following five characteristics have been identified to be representative of the participants (sexually active individuals):

- Number of susceptible individuals (population size and growth)
- Gender
- Age
- Ethnicity
- Self-defined sexual identity

The following five behaviours have been identified as representative of situation (sexual mixing):

- Sexual activity (rate of change of sexual partners)
- Concurrent sexual partnerships
- At least one sexual partner from outside the UK
- Paid for sex
- Frequency of unprotected sex

Individual characteristics and sexual mixing behaviours are relevant to the ecological validity of modelling sexually transmitted infections. For the normative outcomes of cost-effectiveness of universal HPV vaccination to be generalisable and inform public health policies, claims need to be established for the representative design of their underlying models. Ex ante, valid inferences should only be drawn by models which include all the eleven dimensions relevant to individuals and their sexual partner network. Ex post, the relative degree of representativeness of cost-effectiveness studies can be assessed by the number of relevant characteristics and behavioural patterns taken into consideration by their underlying models.

### Study rationale and objectives

The aim of this review is to test the ecological validity of the cost-effectiveness models of universal HPV vaccination available in the literature.

Ecological validity refers to the relation between real-world phenomena and the investigation of these phenomena in experimental contexts or models. This can be framed by asking if the models provide an acceptable representation of the intended reality on all relevant dimensions. In the context of sexually transmitted diseases, patterns of sexual behaviour have been traditionally characterised by focusing on the individual as unit of analysis, e.g. by measuring an individual’s number of sexual partners. However, adopting a network approach to the epidemiology of sexually transmitted infections reveals that behaviours and sexual preferences may influence the spread of infection.

For results to be generalisable, not only should attributes of participants be representative but also their expected behaviour in the sexual mixing.

The review seeks to answer the following research questions:

- From the perspective of ecological validity, are the economic models included in the review representative of real-life sexual attitudes and lifestyles?
- To what degree would an ecological bias threaten the generalisability of the outcomes?
- What are the possible implications on public health policies?

## Methods

### Protocol

The protocol is registered in the International Prospective Register of Systematic Reviews (PROSPERO) CRD42016034145, available at: http://www.crd.york.ac.uk/PROSPERO/display_record.asp?ID=CRD42016034145

The current stage of the systematic review, as defined by PROSPERO, is:

2. Piloting of the study selection process.

### Eligibility criteria

Inclusion criteria are as follows:

1. Studies reporting the incremental cost-effectiveness ratio (ICER) per quality-adjusted life year (QALY) gained of adding males to a female-only HPV vaccination
2. Health outcomes not limited to cervical cancer and genital warts but including additional HPV-induced diseases, such as:
  Vulvar cancer
  Vaginal cancer
  Anal cancer
  Penile cancer
  Oropharyngeal cancer
  Recurrent respiratory papillomatosis (RRP)
3. Studies will be included if HPV universal vaccination will be compared to:
  Cervical cancer screening
  Vaccination of females only
4. Studies reporting a full disclosure of the inputs chosen to inform the economic model
5. Types of economic models:
  Individual models
  Static models
  Transmission dynamic models
  Hybrid models

Exclusion criteria:

1. Health outcomes limited to the valuation of cervical cancer and genital warts
2. Studies not published in English language

### Information sources

The following bibliographic databases will be searched: MEDLINE (via PubMed); Scopus; Science Direct; EMBASE (via OVID SP); Web of Science; EconLit (via EBSCO); DARE, NHIR EED and HTA (via CRD); and CINHAL Plus. An additional search for grey literature will be conducted on Google Scholar and Open Grey. Reviews will be included to reduce the possibility of missing relevant articles.

### Search strategy

A search strategy will be developed for each of the main databases. Additional file 2 reports details of our planned bibliographic database search strategies for MEDLINE (via PubMed); Science Direct; EMBASE (via OVID SP); Web of Science; Scopus; CRD; CINHAL Plus; EconLit (via EBSCO); Google Scholar; and Open Grey.

The PubMed “related articles” search feature will be used to reduce the risk of missing relevant articles. References of the included studies will be searched in order to identify additional relevant studies missed. A pilot of the study selection process will be conducted before initiating the systematic search of relevant articles.

### Study selection

Two graduate research associates (GRAs) will independently identify through database searching, screen, assess for eligibility and include in the review the relevant studies. Opinion of one of the reviewers will be sought to arrive at a consensus in case of disagreement on a study for inclusion.

### Data extraction

Data will be extracted and recorded independently by two additional graduate research associates (GRAs) from the studies included in the review, according to a predefined data extraction table reported in Additional file 3.

Data extracted will be clustered and prioritised: the main outcomes will report the inputs to the demographic and epidemiological model, while additional outcomes will refer to basic inputs to the cost-effectiveness valuation.

Monetary values in different currencies will be transformed in US dollars using purchasing power parities (PPPs). Subsequently, all the monetary values will be adjusted to 2015 dollar value using the US consumer price index (CPI). Corresponding authors of various studies may be contacted by email to clarify methods and results if the need arises.

### Risk of bias

Two external experts (a health economist and a clinical oncologist) will independently assess the risks and threats to validity relative to each of the studies included in the review. The critical appraisal of included economic evaluations will be carried out in two subsequent stages:

- A preliminary stage, aimed to assess the risk of bias in the estimates of treatment effect (e.g. vaccine efficacy) used as data inputs in the economic evaluation. The effectiveness of the HPV vaccines currently available has been preliminarily evaluated by the randomized clinical trials which supported regulatory approval [29]. Vaccines effect and impact of immunisation programmes have been confirmed by post-licensure observational studies [30]. The choice of the appropriate tool to assess risk of bias should reflect relevant differences in the study design. Based on the premise that both randomized clinical trials and observational studies could be equally useful to inform the estimates of relative treatment effect in cost-effectiveness models, the risk of bias will be assessed by using one of the following tools:
  - The Cochrane Risk of Bias tool [31] for randomized clinical trials, available online at http://handbook.cochrane.org/index.htm#chapter_8/figure_8_6_c_example_of_a_risk_of_bias_summary_figure.htm
  - The Cochrane ROBINS-I tool (Risk Of Bias In Non-randomized Studies – of Interventions) [32] for observational studies. The ROBINS-I tool is available online at: https://sites.google.com/site/riskofbiastool/welcome/home
- A main stage, aimed to identify additional risks of bias and, ultimately, to assess the validity of the included studies. The risk assessment will follow the Consolidated Health Economic Evaluation Reporting Standards (CHEERS) approach [33]. The CHEERS statement is available online at: http://www.equator-network.org/wp-content/uploads/2013/04/Revised-CHEERS-Checklist-Oct13.pdf.

In case of misalignment of views, differences will be resolved in a dedicated meeting among reviewers.

### Data synthesis

Each study included in the review will be scored by the number of representative characteristics and behaviours taken into consideration (yes or no) on both dimensions. Individual study’s scores will be plotted in a 2 by 2 matrix [34] reported in Additional file 4.

The upper right quadrant represents the ideal situation, in which both individuals’ characteristics and their sexual partner networks are representatives; hence, the studies are ecologically valid. In the upper left quadrant, we have models describing representative individuals but not in a representative network. In the lower left quadrant, the network is representative but the individuals are not. Finally, in the lower right quadrant, neither the individuals nor the network are representative. Only models in the upper right quadrant provide normative economic recommendations that are fully representative, hence generalisable to inform public health policies.

## Discussion

This protocol describes a systematic review of cost-effectiveness evaluations of universal vaccination programmes against HPV. The primary aim of this review is to test the ecological validity of these models, defined as the degree of representativeness of the individuals susceptible to infection and their sexual preferences and lifestyles.

Eventual gaps identified in this systematic review would have a significant impact on current public health policies. Models in the extant literature seem to converge on the cost-effectiveness of high coverage (>80%) of a single cohort of 12-year-old girls. This vaccination strategy should provide an adequate level of indirect protection (herd immunity) to the unvaccinated boys. This argument presupposes the ecological validity of the cost-effectiveness models; the implicit condition that the characteristics of the individuals and the sexual behaviours observed in the models is generalisable to the natural behaviours of the population. If the ecological validity condition is violated, an error in estimating the protective impact of herd immunity would occur. Hence, a vaccination policy informed on ecologically invalid models would potentially expose boys to a residual risk of contracting HPV-induced malignancies.

## Additional files

Additional file 1: PRISMA-P 2015 checklist. List of recommended items to address in a systematic review protocol. (DOC 82 kb)
Additional file 2: Search strategies. The data provided shows the comprehensive search strategy for the main bibliographic databases. (DOCX 15 kb)
Additional file 3: Data extraction table. The table identifies the specific information to extract for each data category chosen to inform the systematic review. (DOCX 18 kb)
Additional file 4: Data synthesis matrix. The matrix provides a graphical representation of the ecological validity of each cost-effectiveness model included in the systematic review. (PPTX 67 kb)

## Abbreviations

CHEERS: Consolidated Health Economic Evaluation Reporting Standards
CINHAL: Cumulative index to nursing and allied health literature
CPI: Consumer price index
CRD: Centre for reviews and dissemination
DARE: Database of abstracts of reviews and effects
EED: Economic evaluation database
GRA: Graduate research associate
HPV: Human papillomavirus
HTA: Health technology assessment
ICER: Incremental cost-effectiveness ratio
NIHR: National Institute for Health Research
PPP: Purchasing power parities
PRISMA: Preferred reporting items for systematic reviews and meta-analyses
PRISMA-P: Preferred reporting items for systematic review and meta-analysis protocols
PROSPERO: International prospective register of systematic reviews
QALY: Quality-adjusted life years
RRP: Recurrent respiratory papillomatosis

## References

[1] Dunne E, Markowitz L (2006). Genital human papillomavirus infection. Clin Infect Dis.

[2] Baio G, Capone A, Marcellusi A, Mennini FS, Favato G (2012). Economic burden of human papillomavirus-related diseases in Italy. PLoS ONE.

[3] Newall TA, Beutels P, Wood JG, Edmunds WJ, Macintyre CR (2007). Cost-effectiveness analyses of human papillomavirus vaccination. Lancet Infect Dis.

[4] Koleva D, De Compadri P, Padula A, Garattini L (2011). Economic evaluation of human papilloma virus vaccination in the European Union: a critical review. Intern Emerg Med.

[5] Brisson M, Van de Velde N, Boily MC (2009). Economic evaluation of human papillomavirus vaccination in developed countries. Public Health Genomics.

[6] Yahia MCBH, Jouin-Bortolotti A, Dervaux B (2015). Extending the human papillomavirus vaccination programme to include males in high-income countries: a systematic review of the cost-effectiveness studies. Clin Drug Investig.

[7] Audisio RA, Icardi G, Isidori AM, Liverani CA, Lombardi A, Mariani L (2015). Public health value of universal HPV vaccination. Crit Rev Oncol/Haematol.

[8] Marsh K, Chapman R, Baggaley RF, Largeron N, Bresse X (2014). Mind the gaps: what’s missing from current economic evaluations of universal HPV vaccination?. Vaccine.

[9] Centers for Disease Control and Prevention (CDC). HPV vaccine is recommended for boys. December 2nd, 2015. Available online at: http://www.cdc.gov/features/hpvvaccineboys/.

[10] Shamseer L, Moher D, Clarke M, Ghersi D, Liberati A, Petticrew M (2015). PRISMA-P Group. Preferred reporting items for systematic review and meta-analysis protocols (PRISMA-P) 2015: elaboration and explanation. BMJ.

[11] Shmuckler MA (2001). What is ecological validity? A dimensional analysis. Infancy.

[12] Campbell DT (1957). Factors relevant to validity of experiments in social settings. Psychol Bull.

[13] Brunswik E (1956). Perception and the representative design of experiments.

[14] Chapanis A (1961). Men, machines and models. Am Psychol.

[15] Ghani AC, Garnett GP (1998). Measuring sexual partner networks for transmission of sexually transmitted diseases. J R Statist Soc.

[16] Garnett GP, Holmes EC (1996). The ecology of emergent infectious disease. Bioscience.

[17] Fenton KA (2001). Sexual behaviour in Britain: reported sexually transmitted infections and prevalent genital Chlamydia trachomatis infection. Lancet.

[18] Aral SO (2000). Patterns of sexual mixing: mechanisms for or limits to the spread of STIs?. Sex Transm Infect.

[19] The UK Collaborative Group for HIV & STI Surveillance (2007). Testing times. HIV and other sexually transmitted infections in the United Kingdom.

[20] Morris M, Kretzschmar M (1995). Concurrent partnerships and transmission dynamics in networks. Soc Networks.

[21] Watts CH, May RM (1992). The influence of concurrent partnerships on the dynamics of HIV/AIDS. Math Biosci.

[22] Darwich L, Cañadas MP, Videla S, Coll J, Molina-López RA, Sirera G (2013). Prevalence, clearance, and incidence of human papillomavirus type–specific infection at the anal and penile site of HIV-infected men. Sex Transm Dis.

[23] DiClemente RJ, Wingood GM, Crosby RA, Sionean C, Cobb BK, Harrington K (2002). Sexual risk behaviors associated with having older sex partners: a study of black adolescent females. Sex Transm Dis.

[24] Matteelli A, Capone S. The Holy Grail of sexually transmitted infections in travellers. Sex Transm Infect. 2016;92:405–06. 10.1136/sextrans-2016-052573 27272531

[25] Jones KG, Johnson AM, Wellings K, Sonnenberg P, Field N, Tanton C (2014). The prevalence of, and factors associated with paying for sex among men resident in Britain: findings from the third National Survey of Sexual Attitudes and Lifestyles (Natsal-3). Sex Transm Infect.

[26] Garnett GP, Hughes JP, Anderson RM, Stoner BP, Aral SO, Whittington WL (1996). Sexual mixing patterns of patients attending sexually transmitted diseases clinics. Sex Transm Dis.

[27] Aicken CRH, Gray M, Clifton S, Tanton C, Field N, Sonnenberg P (2013). Improving questions on sexual partnerships: lessons learned from cognitive interviews for Britain’s Third National Survey of Sexual Attitudes and Lifestyles (“Natsal-3”). Arch Sex Behav.

[28] Mercer CH, Tanton C, Prah P, Sonnenberg P, Clifton S, Macdowall W (2013). Changes in sexual attitudes and lifestyles in Britain through the life course and over time: findings from the National Surveys of sexual Attitudes and Lifestyles (Natsal). Lancet.

[29] Schiller JT, Castellsagué X, Garland SM (2012). A review of clinical trials of human papillomavirus prophylactic vaccines. Vaccine.

[30] Garland SM, Kjaer SK, Muñoz N, Block SL, Brown DR, DiNubile MJ (2016). Impact and effectiveness of the quadrivalent human papillomavirus vaccine: a systematic review of 10 years of real-world experience. Clin Infect Dis.

[31] Higgins JPT, Altman DG, Gøtzsche PC, Jüni P, Moher D, Oxman AD (2011). The Cochrane Collaboration’s tool for assessing risk of bias in randomised trials. BMJ.

[32] Sterne JAC, Hernán MA, Reeves BC, Savović J, Berkman ND, Viswanathan M (2016). ROBINS-I: a tool for assessing risk of bias in non-randomised studies of interventions. BMJ.

[33] Husereau D, Drummond M, Petrou S, Caarswell C, Moher D, Greemberg D (2013). Consolidated Health Economic Evaluation Reporting Standards (CHEERS)—explanation and elaboration: a report of the ISPOR Health Economic Evaluation Publication Guidelines Good Reporting Practices Task Force. Value Health.

[34] Hogarth MR (2005). The challenge of representative design in psychology and economics. J Econ Methodol.

