# Supplementary material for: Ecological validity of cost-effectiveness models of universal HPV vaccination: a protocol for a systematic review
Source: Syst Rev. 2017 Jan 25;6:17. doi: 10.1186/s13643-017-0409-7 (PMC5264325; doi:10.1186/s13643-017-0409-7)
Supplement: Additional file 2: — Search strategies. The data provided shows the comprehensive search strategy for the main bibliographic databases. (DOCX 15 kb) [file 13643_2017_409_MOESM2_ESM.docx]

# Additional file 2: search strategies by bibliographic database

# Medline (via PubMed)

(((((((boy[All Fields] OR ("men"[MeSH Terms] OR "men"[All Fields])) OR ("male"[MeSH Terms] OR "male"[All Fields])) AND HPV[All Fields]) OR ("papillomaviridae"[MeSH Terms] OR "papillomaviridae"[All Fields] OR "papillomavirus"[All Fields])) AND ("vaccination"[MeSH Terms] OR "vaccination"[All Fields])) AND ("economics"[Subheading] OR "economics"[All Fields] OR "cost"[All Fields] OR "costs and cost analysis"[MeSH Terms] OR ("costs"[All Fields] AND "cost"[All Fields] AND "analysis"[All Fields]) OR "costs and cost analysis"[All Fields])) AND effectiveness[All Fields]) AND model[All Fields]

# Science Direct

"boy" OR "men" OR "male" AND "HPV" OR "papillomavirus" AND "vaccination" AND "cost" AND "effectiveness" AND "model" AND LIMIT-TO(**topics, "hpv vaccination"**).

# Embase via OVID SP

HPV or human papillomavirus) AND vaccination AND cost effectiveness AND (boys or males or men)

# WEB OF SCIENCE

**TOPIC**: (HPV OR papillomavirus) **AND TOPIC**: (economic and evaluation and model) **AND TOPIC**: (universal or male or boy or men) **AND TOPIC**: (vaccination)

**Timespan**: All years.

Search language=English

# Scopus

( TITLE-ABS-KEY ( **boy** ) OR TITLE-ABS-KEY ( **men** ) OR TITLE-ABS-KEY ( **male** ) AND TITLE-ABS-KEY ( **hpv** ) OR TITLE-ABS-KEY ( **papillomavirus** ) AND TITLE-ABS-KEY ( **vaccination** ) AND TITLE-ABS-KEY ( **cost** ) AND TITLE-ABS-KEY ( **effectiveness** ) AND TITLE-ABS-KEY ( **model** ) ) AND DOCTYPE ( **ar** OR **re** ) AND SUBJAREA ( **mult** OR **agri** OR **bioc** OR **immu** OR **neur** OR **phar** OR **mult** OR **medi** OR **nurs** OR **vete** OR **dent** OR **heal** )

# CRD

((boy OR men OR male) AND (HPV OR papillomavirus) AND (vaccination)) and ((Systematic review:ZDT and Bibliographic:ZPS) OR (Systematic review:ZDT and Abstract:ZPS) OR (Cochrane review:ZDT) OR (Cochrane related review record:ZDT) OR (Economic evaluation:ZDT and Bibliographic:ZPS) OR (Economic evaluation:ZDT and Abstract:ZPS) OR Project record:ZDT OR Full publication record:ZDT) IN DARE, NHSEED, HTA

# CINAHL Plus

TX ( boy or men or male ) AND TX ( hpv or human papillomavirus ) AND TX cost effectiveness AND TX model

# EconLit via EBSCO

Hpv or human papillomavirus) AND vaccination

# Google Scholar

allintitle: HPV OR papillomavirus AND vaccination AND boy OR men OR male

# Open Grey

Human papillomavirus vaccination lang:"en"
