# Supplementary material for: Ecological validity of cost-effectiveness models of universal HPV vaccination: a protocol for a systematic review
Source: Syst Rev. 2017 Jan 25;6:17. doi: 10.1186/s13643-017-0409-7 (PMC5264325; doi:10.1186/s13643-017-0409-7)
Supplement: Additional file 3: — Data extraction table. The table identifies the specific information to extract for each data category chosen to inform the systematic review. (DOCX 18 kb) [file 13643_2017_409_MOESM3_ESM.docx]

**Additional file 3: data extraction table**

| Data category | Specific information to extract |
| --- | --- |
| *Inputs to demographic and epidemiological model* |  |

Demographic model Structure, growth and evolution of the observed population (e.g. stable; stationary; zero, geometric, exponential or logistic growth; open or closed; age-structured multiple cohorts)

Population size The population upon which the results of the analysis can be reasonably applied (e.g. the number of sexually active individuals observed at any given point in time)

Time horizon The length of time in which resource use and health effects are measured (years)

Gender Stratification of the study population by gender (male/female)

Age Stratification of the study population by years of age

Ethnicity Stratification of the study population by main ethnic group

Sexual identity Self-defined sexual identity (e.g. heterosexual or homosexual)

Sexual activity Rate of change of sexual partners over a defined period of time (e.g. x new partners in y years)

Concurrent partnerships Frequency of overlapping sexual partnerships

Sexual partners from abroad Frequency of sexual partners travelling abroad or born outside the population observed

Paid for sex Frequency of paid sexual intercourses

Unprotected sex Frequency of sexual intercourses performed without the use of condoms

|  |  |
| --- | --- |
| *Inputs to cost effectiveness analysis* |  |

Model Model design (e.g. static, dynamic, hybrid)

Perspective National Health System, patients or societal perspective

Country of study The Country (or Countries) for which the study and results are applicable

Economic inputs Costs and benefits included in the analysis (e.g. direct, indirect, both)

Intervention compared Vaccination and/or screening strategies compared (e.g. 12 year old male and females vs. 12 year old female only)

Vaccine coverage Percent of the targeted population receiving vaccination

Vaccine efficacy Percentage reduction of disease in a vaccinated group of people compared to an unvaccinated group

Duration of protection Years of immunity provided by vaccination

Disease classification The primary disease(s) addressed by the intervention and the comparator (e.g. genital warts, cervical, vulvar, vaginal cancer, penile, oropharyngeal cancer and respiratory papillomatosis)

Vaccine price Price per vial (local currency, unadjusted)

Discount rate The rate used to compute present value of costs and benefits incurred

ICER/QALY gained Incremental Cost Effectiveness ratio (ICER): the difference in cost between two possible interventions, divided by the difference in Quality Adjusted Life Years (QALY) gained (local currency, unadjusted)

Year based for currency value Year when the analysis (both costs and benefits) was conducted

Sensitivity analysis Parameters that had the highest impact on ICER (when reported)

Acceptability threshold Threshold used by the authors of the study to determine if the intervention studied is cost-effective and/or should be adopted
