# Supplementary material for: Ecological validity of cost-effectiveness models of universal HPV vaccination: a protocol for a systematic review
Source: Syst Rev. 2017 Jan 25;6:17. doi: 10.1186/s13643-017-0409-7 (PMC5264325; doi:10.1186/s13643-017-0409-7)
Supplement: Additional file 4: — Data synthesis matrix. The matrix provides a graphical representation of the ecological validity of each cost-effectiveness model included in the systematic review. (PPTX 67 kb) [file 13643_2017_409_MOESM4_ESM.pptx]

## Slide 1
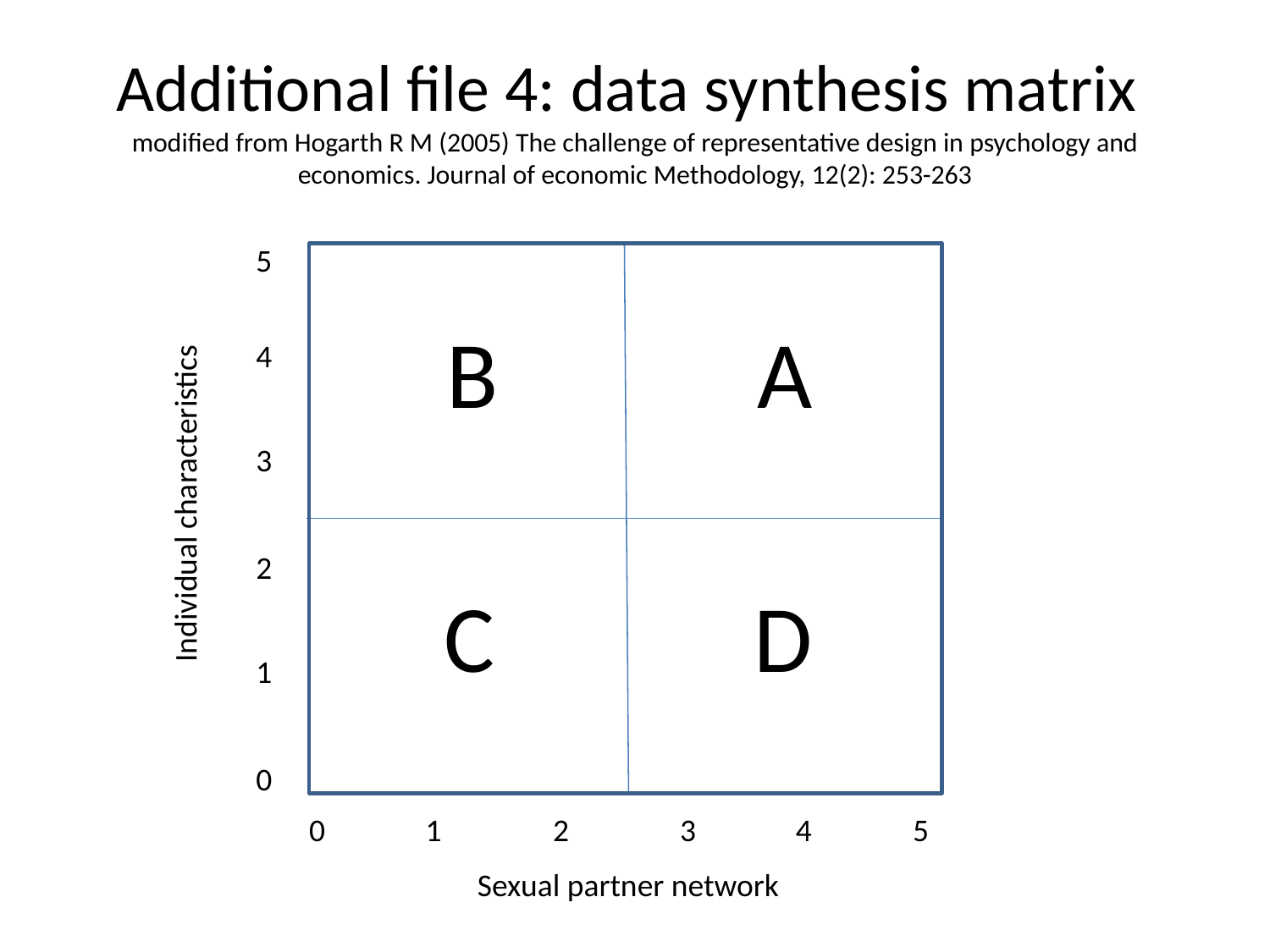

# Additional file 4: data synthesis matrix modified from Hogarth R M (2005) The challenge of representative design in psychology and economics. Journal of economic Methodology, 12(2): 253-263
5
B
A
4
3
Individual characteristics
2
C
D
1
0
0
1
2
3
4
5
Sexual partner network
